# The Relationship between Obesity and Urban Environment in Seoul

**DOI:** 10.3390/ijerph14080898

**Published:** 2017-08-09

**Authors:** Jungah Kim, Changwoo Shon, Seonju Yi

**Affiliations:** 1 The Seoul Institute, 57 Nambusunhwan-ro, 340-gil, Seocho-gu, Seoul 06756, Korea; 0826kja@naver.com (J.K.); cwshon@si.re.kr (C.S.); 2 Graduate School of Public Health, Seoul National University, 1 Gwanak-ro, Gwanak-gu, Seoul 08826, Korea

**Keywords:** obesity, physical activity, food environment, urban, multilevel analysis

## Abstract

Obesity is a global pandemic that brings about a myriad of health consequences. In the past, policies for combating obesity mainly focused on improving individual health and behavior, but nowadays some policies have changed and now concentrate on improving the built environment believing this can improve health through positive changes to health-related behaviors. We examined whether both individual and environmental factors were associated with body mass index in Seoul, the capital city of South Korea. Data from the 2011 and 2013 Community Health Surveys were used (*n* = 20,147 men and 25,300 women). We staged multilevel logistic regression models to estimate the effect of individual and environmental factors on obesity. Among individual covariates, high-risk drinking, the time spent watching TV and surfing the Internet, high salt intake, stress, and the negative recognition of health were significantly associated with obesity. When controlling individual covariates, the number of sports facilities, number of fried chicken stores, and food insecurity level were statistically associated with probability of obesity. Therefore, this study emphasizes that it is important not only to improve the health behavior of the individual, but also to improve the urban environment in order to reduce the obesity rates of city dwellers.

## 1. Introduction

Obesity, one of the largest health risk factors, is of great concern internationally [1]. The worldwide prevalence of obesity has nearly doubled since 1980, according to the World Health Organization [2]. Obesity results in a host of health consequences such as cardiovascular disease, diabetes, hypertension, and causes social consequences including depression and psychological pain [3,4].

In South Korea, the obesity rate in 2015 was 33.2%, which resulted in an astronomical figure of socioeconomic costs of up to $55 billion in 2013, 2.2 times higher compared to 2005 [5]. In Seoul, the capital of South Korea, the obesity rate was not high compared to other megacities in Western countries such as New York, however, the rate has consistently increased from 20.7% in 2008 to 23.6% in 2014 [6], and this was the highest obesity rate among Asian countries. Moreover, there were differences in obesity rate between men and women where men’s obesity rate was 39.7%, higher than the 26% of women in 2014, and disparities among administrative districts were also highlighted as a problem in terms of obesity in Seoul.

In the past, policies combating obesity in Seoul usually focused on improving individual health behaviors, reducing food intake and increasing physical activities, and educating about health promotion. These policies were provided through health centers in each administrative district; however, the obesity rate has constantly increased, reflecting the ineffectiveness of these policies. In this context, there has been a global surge in the emphasis of the impact of built environment on obesity and comprehensive approaches have emerged since the 2000s, such as the ecological model of health determinants, considering factors from the individual to environmental level to prevent obesity [7,8,9,10,11,12,13,14,15,16,17,18,19]. That is, this concept supposes that the built environment and lifestyle of urban residents affects their health status and one of the assumptions is that unexpected health problems have appeared due to rapid global urbanization. In line with the ecological model, Health City projects have been started in several cities in Europe that try to change the health environment by finding health determinants and coordinating and intervening in relevant policies [20]. The Seoul Metropolitan government joined the Alliance for Health Cities (AFHC) in 2004, and now 23 out of 25 administrative districts participate as Health City members; however, the aim of Health City didn’t feed into urban planning in either Seoul or each administrative district.

As a result of increasing urbanization, people have no choice but to live in confined spaces, and in addition, environments that decrease the physical activities of urban residents have appeared, and car usage has increased as the distance between residences and workplaces has widened [21,22]. Old downtown areas usually fall short of pedestrian amenities and open spaces, and these areas usually have poor street patterns. Further, an unhealthy food environment, such as easy access to high-calorie foods and negative perception of nonhomogeneous neighborhoods, makes urban residents likely to be obese [23,24]. Seoul also has a harmful environment to health because the population density is very high and open spaces are limited. Regarding the food environment, there are many fast food stores and street food establishments, and people can order any kind of food through 24-h delivery services at any time.

As previously mentioned, the obesity rate of Seoul has steadily increased, and the disparities among administrative districts have grown. Against the backdrop of this situation, the present study was conducted to provide the grounds for building a healthy environment in Seoul and it explored both the individual and environmental determinants of obesity in Seoul based on the ecological model of health.

## 2. Materials and Methods

### 2.1. Design and Participants

Data on our sample of respondents (persons aged 19 years or older) were derived from Community Health Surveys in 2011 and 2013 for obesity and individual covariates. The Community Health Survey is an ongoing visiting survey of residents in South Korea and is continuously conducted annually between August and October. The target population included all residents living in South Korea and the target sample included residents living in a residence such as an apartment or house at each sampling location. Respondents were asked questions from modules on demographics, health behaviors, health status, accessibility to health services, incidents and addiction, and quality of life. In terms of research ethics, The Community Health Survey has been annually reviewed and approved by the Korea Centers for Disease Control and Prevention since 2010. In this research, we used data from residents to examine the determinants of obesity in Seoul citizens and the target sample comprised 20,147 men and 25,300 women over 2 years. Data from the Seoul Survey, Employer Basic Survey, Freedom of information and Transportation Society, Seoul Employer Survey, Clean plus website, Seoul Metropolitan Police Agency, and Health Insurance Review and Association Service (HIRA) were also used for environmental-level variables.

### 2.2. Measurements

Body mass index (BMI; weight in kilograms divided by height in meters squared) is based on self-reported height and weight. According to the definition of the World Health Organization for the Western Pacific Region (WPRO), we classified individuals with BMI of 25 kg/m^2^ or more as obese and those with a BMI less than 25 kg/m^2^ as non-obese [25,26]. Thus, the outcome variable in our study was whether a person was obese or not; we regarded a BMI over 25 as 1 and a BMI below 25 as 0 (dummy variable).

#### 2.2.1. Individual-Level Variables

We collected data from the Community Health Survey and the following individual-level variables were included in Model 2 and Model 4: For socio-demographic variables, age groups were divided at 10-year intervals (20–29, 30–39, 40–49, 50–59, 60–69, over 70), and household income groups were classified as five quintiles where the first group was the lowest income group and the fifth group was the highest income group. With regard to educational attainment, the participants were asked the level at which their education was completed, which was classified into four educational categories: lower than middle school, high school graduate, college graduate, and graduate school or higher.

For health behavior, current smoking status (whether respondents smoked or not), high-risk drinking (yes/no based on adequate drinking), and drinking period were asked. High-risk drinking referred to men drinking over seven glasses of beer or women drinking over five glasses of beer at one drinking party for more than two days a week. Walking rate was also included in this study, i.e., whether respondents walked over 30 min for more than 5 days in a week or not, as well as the time people spent watching TV and surfing the Internet during leisure time in the last week. We divided people according to whether they spent more/less than 3 h a day watching TV and surfing the Internet. For vegetable and fruit intake, we classified respondents according to whether they consumed fruits or vegetables more than once in a day in the last one month or not, and asked regarding high salt intake in daily life based on yes/no. For health status, we asked whether one was stressed during daily life, and used a measure of self-reported health in which a person recognized him/herself during daily life based on good/bad.

#### 2.2.2. Environmental-Level Variables

We divided environmental-level variables into three categories: physical activity environment, food environment, and urban environment. Physical activity environment variables included the area of parks in a person’s living spheres, number of physical training centers per 10,000 persons, rate of commute by cars, and satisfaction with walking environment. The area of parks in a person’s living spheres in the Seoul Survey is calculated by dividing the sum of the areas of urban nature parks, neighborhood parks, children’s parks, mini-parks, sport parks, culture parks, historic parks, and waterside parks (meters squared) by population. Satisfaction with walking environment in the Seoul Survey is the degree of satisfaction with walking in the neighborhood and downtown Seoul, which ranged from 0 to 10 points.

Food environment variables included the food insecurity index, number of fast food stores per 10,000 persons from the Freedom of Information and Transparent Survey, and number of fried chicken stores per 10,000 persons from the Seoul Employer Survey. The food insecurity index, in the Seoul Survey, is the rate of people who answered, “I often fell short of food due to economic burden in the most recent one year”.

Urban environment variables included urbanization rate, social trust, fiscal self-reliance ratio, crime rate, and the number of beds per 10,000 persons. Urbanization rate, from Seoul Statistics, is calculated by dividing the sum of residential, commercial, and manufacturing areas out of use districts by the area of administrative districts. Social trust, from the Seoul Survey, is the degree of trust for family, neighborhood, complete strangers, foreigners, and government offices, which ranged from 0 to 10. Fiscal self-reliance ratio, from the Clean Plus website, was calculated by dividing one’s income (sum of local taxes and non-tax receipts) by the size of the general accounting budget. Crime rate, from the Seoul Metropolitan Police Agency, was calculated by dividing the number of violent crimes (murder, robbery, rape, larceny, violence) by 100,000 persons. For the number of beds per 10,000 persons, the source was from Health Insurance Review and Assistance sService, and the beds of hospitals and clinics were included in the calculation.

### 2.3. Data Analysis

Multilevel analysis has emerged as an analytical strategy that allows the simultaneous examination of group-level and individual-level factors. The use of multilevel analysis raises theoretical and methodological issues related to the theoretical model being tested, conceptual distinction between group- and individual-level variables, ability to differentiate “independent” effects, reciprocal relationships between factors at different levels, and the increased complexity that these models apply [27]. As mentioned earlier, there were obesity disparities among administrative districts in Seoul, thus, multilevel logistic regression was used to estimate the determinants of obesity measured at the individual and environmental level to comprehend the reasons for these disparities. Four models were staged for each outcome: Model 1, the null model, did not contain any covariates in order that both the individual and environmental level variance in the outcomes could be assessed in the absence of any explanatory variables. Model 2 contained only the individual-level covariates; Model 3 contained only the environmental-level covariates; and finally, Model 4 contained the individual-level and environmental-level covariates. A model for these estimation methods is described in the following equation where $Y_{ij}$ is obesity, $X_{ij}$ are individual i’s characteristics residing in j district, and $Z_{j}$ are environmental characteristics of j district:

$$\mathrm{logit}\left\{P_{r}\left(Y_{ij}=1|X_{ij},\;Z_{j}\right)\right\}=\gamma_{00}+\gamma_{10}X_{ij}+\gamma_{01}Z_{j}+\gamma_{11}X_{ij}Z_{J}+U_{1j}X_{ij}+U_{0J}+\varepsilon_{ij}$$

## 3. Results

### 3.1. Participants’ General Characteristics

Descriptive statistics of the study sample are provided in Table 1. In total, 45,447 Seoul citizens were included in the study, which included 20,147 men and 25,300 women. Age and household income groups of respondents were evenly distributed. For educational attainment, a large majority of the sample was high school-graduated and college-graduated. More male respondents (40.4%) were smokers than female respondents (3.5%). Nearly half of the respondents among both men (56.6%) and women (53.3%) walked more than 5 days a week for a total of 30 min or more per day. Over 70% of the respondents did not watch the television or surf the Internet more than 3 h in a day, replied that they were not stressful, and thought themselves as healthy people. More female respondents (58.9%) consumed fruits than male respondents (43.7%). More respondents did not eat vegetables or high salt foods than those who did. The overall obesity prevalence of the sample was 29.8% for men and 16.7% for women.

### 3.2. Multilevel Analyses

Individual and environmental factors associated with BMI are shown in Table 2 and Table 3. Model 2 shows the associations between obesity and individual factors including sociodemographic characteristics, health behavior, and health status. For men, age, income, education attainment, smoking, high-risk drinking, drinking period, walking, high salt intake, stress, and self-reported health were associated with obesity. For women, age, income (only for the 5th quintile), education attainment, smoking, high-risk drinking, time spent watching TV and surfing the Internet, fruit intake, high salt intake, stress, and self-reported health had associations with obesity. Model 3 shows the influence of environmental factors on obesity. For men, the number of physical training centers was significantly associated with obesity whereas the number of fast food stores was significant for women. Model 4 shows the correlations between obesity and factors from the individual to environmental level.

For men, age, education attainment, time spent watching TV and surfing the Internet, high-risk drinking, high salt intake, stress, self-reported health, and the number of fried chicken stores significantly increased the likelihood of being obese. Particularly male respondents in their 30s and 40s and those whose income was over the 3rd quintile (4th and 5th quantile) were more likely to be obese whereas men older than 70 had lower chances of obesity. However, smoking, walking, number of physical training centers, and food insecurity rate were negatively associated with obesity. For women, age, time spent watching TV and surfing the Internet, high-risk drinking, high salt intake, stress, and self-reported health were positively related to being obese. However, income over the 5th quintile, education attainment, smoking, and fruit intake significantly decreased obesity in women. In other words, women with higher education levels had a lower possibility of obesity. The results showed that women were not affected by environmental factors, unlike men.

## 4. Discussion

The main purpose of our study was to explore both the individual and environmental determinants of obesity in Seoul based on the social ecological model of obesity. When controlling individual covariates, for men, the number of sports facilities was associated with obesity. Considering that sports facilities are the places that encourage people to exercise, the higher the number of sports facilities in administrative districts there were, the lower the probability of men’s obesity [18,28,29]. This result was also related with urban characteristics, where most men commuted across the districts and tried to find places for exercise after work. In Seoul, there are many gyms where people can exercise safely until dawn in any weather.

Further, we demonstrated that the number of fried chicken stores was associated with obesity; the more fried chicken stores there were, the higher the obesity probability. There were no earlier studies that estimated the impact of fried chicken stores on obesity, however, fried chicken stores were usually perceived as places similar to fast food stores, i.e., selling high-calorie foods and leading to obesity in South Korea. Thus, our result was in line with earlier studies using fast food stores as a proxy for food environment influences on obesity [9,29,30,31,32], which found that higher restaurant density was associated with higher BMI among local residents. In addition, it is popular in South Korean culture to have fried chicken and drink beer together especially at night when people usually go to restaurants or use food delivery services. Therefore, this culture can support the result of our study; the number of fried chicken stores are associated with obesity.

The correlation between food security and obesity was inconclusive. Recently, studies have suggested that inconsistent access to resources may be partially responsible for the increased prevalence of obesity among individuals in low-income households. For individuals in households with intermediate levels of food insecurity, gradual weight gain could occur from either inconsistent access to food, leading to periods of underconsumption followed by compensatory overconsumption [33,34,35,36,37,38,39], or from consuming inexpensive foods with high energy density when less money is available to spend on food [40,41]. This was in contrast with our finding that higher food insecurity rate statistically decreased obesity probability, and some of the inconsistencies among prior studies might be due to differences in the food culture of regions and subpopulations examined.

Compared to men, the results showed that women were not influenced by environmental factors. These results might be due to the fact women in Seoul usually do not exercise; the women’s walking ratio was 53.5% in 2014, which was lower than men (57.0%). Therefore, the physical environment around women might not have an influence on their physical activities and obesity probability. Regarding the food environment, women might be more sensitive to their own body shape and may be more likely to cook and eat food on their own compared to men. In light of the characteristics mentioned earlier, the obesity ratio of women was mostly low, so there were possibilities of an association between environmental factors and obesity in women. However, the obesity probability of women has grown consistently; therefore, we need to monitor their environmental factors and obesity level continuously.

This study found that, for men, physical environment factors such as the number of sports facilities, number of fried chicken stores had an influence on the obesity prevalence of individuals from the perspective of urban health. In South Korea, the rate of women who exercise intensely for 30 min in a day for more than 5 days (25.9%) is lower than men (17.7%) [6], so for women, the influence of sports facilities on obesity might be smaller than men. Currently, swimming pools funded by Seoul City and administrative districts exist in the Seoul Metropolitan Area, but the economic accessibility of these facilities is low and many people cannot use them. Thus, it is necessary to expand sports facilities, such as fitness centers or swimming pools, at the level of Seoul City and administrative districts across Seoul to improve economic accessibility and make them easier for citizens to use.

In addition, obesity is often influenced by food and beverage policy, taxation, transportation, and especially urban policy with individual responsibility [42]. Therefore, effective management of obesity can be accomplished through cooperation between different departments. Moreover, further research is needed, especially studies with longitudinal designs or based on respondents’ living areas, to determine whether modifications in the environment may aid in curbing the current obesity epidemic.

This study examined the associations between environmental factors and obesity, although it had some limitations. First, our analysis was based on respondents’ residential areas; however, some people spend more time around their work places than residential areas. Thus, there are several possible environmental factors in workplaces that could affect respondents’ obesity more than those of residential areas, which we could not consider due to data limitations. Another possible limitation is a cross-border issue where we collected environmental factors by administrative districts; therefore, some respondents’ life zones could have overlapped. If one person lives in an administrative district close to another administrative district, then his/her life zone will cover two administrative districts. In other words, there remains a possibility that direct environmental effects on individual’s obesity could be somewhat underestimated because the area of an administrative district as the unit for analysis was too broad. An administrative district, however, is the smallest unit to plan and implement health policies, and each administrative district in Seoul establishes its local health care plan every four years. Our study identified health risk factors based on administrative district; therefore, the results could be used as basic data for establishing local health care plans. Lastly, there were issues of data accessibility related to the variables used in our analysis. For some environmental factors such as neighborhood aesthetics (cleanliness), access to convenience stores or supermarkets, and street food access, we could not get data where any official data were not collected for analysis. Thus, the relationships found between the environmental factors and obesity in our study cannot be considered causal. Despite some limitations, this study is the first in Seoul that includes a large spectrum of environmental variables to grasp the impact of community environment on obesity by using extensive administrative data of Seoul in comparison with other studies that considered only one or a few environmental variables. Another strong point of our study is the large sample, which allowed us to understand the effect of environmental factors on obesity in great detail.

## 5. Conclusions

Korean society still considers obesity as a health problem and obese people as being lazy, weak-willed, unsuccessful, and as having poor willpower [43,44]. However, obesity is not solely due to individual behaviors. Obesity may lead to lots of chronic diseases, so it is especially important to prevent and manage obesity in advance. But this study emphasized one of the reasons why obesity has not been solved is the environment. For this, we examined the effects of environmental factors around respondents, such as physical, food, and urban environment on individual obesity through multilevel analysis, and in particular, the effects of a district’s environmental factors in terms of urban health; the results showed that obesity and the living environment was correlated, which was in agreement with previous studies.

## Figures and Tables

**Table 1:** Descriptive characteristics of study sample and obesity prevalence.

Variable	Men		Women	
	*n*	%	*n*	%
Total	20,147	100	25,300	100
Age				
19–29	3300	16.4	4153	16.4
30–39	4353	21.6	5124	20.3
40–49	4201	20.9	5228	20.7
50–59	3633	18.0	4986	19.7
60–69	2691	13.4	3347	13.2
Over 70’s	1969	9.8	2462	9.7
Household income				
First group	4292	21.3	6095	24.1
Second group	3536	17.6	4279	16.9
Third group	4031	20.0	4860	19.2
Fourth group	3733	18.5	4509	17.8
Fifth group	4555	22.6	5557	22.0
Educational attainment				
Lower than middle school	3327	16.5	6553	25.9
High school graduate	7266	36.1	8748	34.6
College graduate	8049	40.0	8994	35.5
Graduate school or higher	1505	7.5	1005	4.0
Current smoking status				
Yes	8130	40.4	881	3.5
No	12,017	59.6	24,419	96.5
Walking				
Yes	11,413	56.6	13,488	53.3
No	8734	43.4	11,812	46.7
Television viewing or internet surfing				
Yes	5276	26.2	6702	26.5
No	14,871	73.8	18,599	73.5
Fruit intake				
Yes	8812	43.7	14,891	58.9
No	11,335	56.3	10,409	41.1
Vegetable intake				
Yes	6711	33.3	9498	37.5
No	13,436	66.7	15,802	62.5
High salt intake				
Yes	6561	32.6	5776	22.8
No	13,586	67.4	19,524	77.2
Stress level				
Non-stressful	14,379	71.4	17,814	70.4
Stressful	5768	28.6	7486	29.6
Self-reported health				
Good	2217	11.0	3969	15.7
Bad	17,930	89.0	21,331	84.3
Obesity				
Low weight	454	2.3	2281	9.0
Normal weight	13,681	67.9	18,785	74.2
Obese	6012	29.8	4234	16.7

**Table 2:** Individual and environmental factors affecting obesity of men in Seoul based on multilevel analysis results.

	Model 1			Model 2			Model 3			Model 4		
	Estimate	S.E.	Pr > |t|	Estimate	S.E.	Pr > |t|	Estimate	S.E.	Pr > |t|	Estimate	S.E.	Pr > |t|
Intercept	−0.854	0.022	<0.0001	−1.520	0.086	**<.0001**	−0.362	0.721	0.624	−0.686	0.699	0.346
Individual-level predictors												
Age, groups (19–29, reference)												
30–39				0.584	0.064	**<0.0001**				0.578	0.064	**<0.0001**
40–49				0.411	0.085	**<0.0001**				0.403	0.085	**<0.0001**
50–59				0.183	0.113	0.104				0.175	0.113	0.122
60–69				−0.019	0.145	0.893				−0.029	0.145	0.840
Over 70’s				−0.459	0.181	**0.011**				−0.475	0.181	**0.009**
Household income (First group, reference)												
Second group				0.018	0.055	0.741				0.016	0.055	0.776
Third group				0.127	0.053	**0.017**				0.124	0.053	**0.020**
Fourth group				0.143	0.055	**0.009**				0.140	0.055	**0.011**
Fifth group				0.112	0.054	**0.039**				0.115	0.054	**0.034**
Educational attainment (Lower than middle school, reference)												
High school graduate				0.017	0.055	0.758				0.018	0.055	0.741
College graduate				0.145	0.057	**0.011**				0.153	0.058	**0.008**
Graduate school or higher				0.190	0.078	**0.015**				0.202	0.079	**0.010**
Current smoking status				−0.188	0.035	**<0.0001**				−0.188	0.035	**<0.0001**
High risk drinking				0.299	0.037	**<0.0001**				0.298	0.037	**<0.0001**
Drinking period				0.008	0.003	**0.026**				0.008	0.003	**0.022**
Walking				−0.080	0.033	**0.014**				−0.081	0.033	**0.014**
Television viewing or internet surfing				0.081	0.038	**0.032**				0.082	0.038	**0.030**
Fruit intake				−0.001	0.035	0.968				−0.002	0.035	0.960
Vegetable intake				−0.015	0.036	0.672				−0.015	0.036	0.668
High salt intake				0.237	0.034	**<0.0001**				0.236	0.034	**<0.0001**
Stress level												
Non-stressful(reference)												
Stressful				0.095	0.036	**0.008**				0.095	0.036	**0.008**
Self-reported health												
Good(reference)												
Bad				0.112	0.056	**0.047**				0.111	0.056	**0.048**
Environment-level predictor												
The area of parks							−0.013	0.012	0.305	−0.011	0.012	0.364
The number of sports facilities							−0.142	0.056	**0.027**	−0.127	0.054	**0.038**
The rate of commute by cars							0.001	0.001	0.330	0.002	0.001	0.100
Satisfaction on walking environment							0.012	0.009	0.203	0.012	0.008	0.170
Food insecurity rate							−0.188	0.091	0.061	−0.217	0.088	**0.029**
The number of fast food stores							−0.012	0.022	0.592	−0.007	0.022	0.765
The number of fried chicken stores							0.363	0.184	0.073	0.393	0.178	**0.048**
Urbanization rate							−0.011	0.051	0.825	−0.040	0.049	0.431
Social trust							0.128	0.071	0.097	0.102	0.069	0.165
Fiscal self-reliance ratio							0.009	0.005	0.136	0.006	0.005	0.273
Crime rate							0.000	0.000	0.159	0.000	0.000	0.462
The number of beds							0.002	0.005	0.654	0.001	0.005	0.778
Random Effects												
$\partial^{2}$	0.007	0.004	0.033	0.005	0.003	0.064	0.003	0.003	0.223	0.002	0.003	0.316

**Table 3:** Individual and environmental factors affecting obesity of women in Seoul based on multilevel analysis results.

	Model 1			Model 2			Model 3			Model 4		
	Estimate	S.E.	Pr > |t|	Estimate	S.E.	Pr > |t|	Estimate	S.E.	Pr > |t|	Estimate	S.E.	Pr > |t|
Intercept	−1.617	0.045	<0.0001	−2.341	0.106	**<0.0001**	−2.617	0.710	0.003	−3.209	0.847	**0.003**
Individual-level predictors												
Age, groups (19–29, reference)												
30–39				0.709	0.085	**<0.0001**				0.702	0.085	**<0.0001**
40–49				0.970	0.088	**<0.0001**				0.964	0.088	**<0.0001**
50–59				1.063	0.098	**<0.0001**				1.062	0.098	**<0.0001**
60–69				1.317	0.112	**<0.0001**				1.317	0.112	**<0.0001**
Over 70’s				1.121	0.134	**<0.0001**				1.121	0.134	**<0.0001**
Household income (First group, reference)												
Second group				0.037	0.061	0.543				0.031	0.061	0.611
Third group				−0.005	0.062	0.936				−0.010	0.062	0.870
Fourth group				−0.069	0.066	0.296				−0.072	0.066	0.272
Fifth group				−0.257	0.068	**0.000**				−0.247	0.068	**0.000**
Educational attainment (Lower than middle school, reference)												
High school graduate				−0.291	0.057	**<0.0001**				−0.280	0.057	**<0.0001**
College graduate				−0.759	0.071	**<0.0001**				−0.732	0.071	**<0.0001**
Graduate school or higher				−1.129	0.152	**<0.0001**				−1.086	0.153	**<0.0001**
Current smoking status				−0.265	0.108	**0.014**				−0.255	0.108	**0.018**
High risk drinking				0.199	0.092	**0.031**				0.199	0.092	**0.030**
Drinking period				0.002	0.002	0.374				0.002	0.002	0.323
Walking				−0.020	0.040	0.617				−0.021	0.040	0.598
Television viewing or internet surfing				0.314	0.044	**<0.0001**				0.311	0.044	**<0.0001**
Fruit intake				−0.091	0.043	**0.033**				−0.088	0.043	**0.040**
Vegetable intake				−0.035	0.043	0.421				−0.031	0.043	0.479
High salt intake				0.323	0.045	**<0.0001**				0.324	0.045	**<0.0001**
Stress level												
Non-stressful(reference)												
Stressful				0.176	0.044	**<0.0001**				0.176	0.044	**<0.0001**
Self-reported health												
Good(reference)												
Bad				0.219	0.055	**<0.0001**				0.218	0.055	**<0.0001**
Environment-level predictor												
The area of parks							−0.019	0.012	0.139	−0.022	0.014	0.153
The number of sports facilities							−0.119	0.057	0.058	−0.076	0.069	0.288
The rate of commute by cars							0.002	0.001	0.157	0.001	0.002	0.449
Satisfaction on walking environment							−0.006	0.009	0.495	−0.009	0.010	0.383
Food insecurity rate							0.049	0.087	0.579	0.136	0.104	0.214
The number of fast food stores							0.062	0.021	**0.014**	0.036	0.026	0.187
The number of fried chicken stores							−0.108	0.181	0.563	0.134	0.217	0.548
Urbanization rate							0.069	0.050	0.193	0.045	0.060	0.466
Social trust							0.086	0.070	0.242	0.018	0.083	0.829
Fiscal self-reliance ratio							0.000	0.005	0.985	−0.001	0.006	0.905
Crime rate							0.000	0.000	0.748	0.000	0.000	0.835
The number of beds							0.001	0.005	0.843	−0.004	0.006	0.589
Random Effects												
$\partial^{2}$	0.044	0.015	0.002	0.014	0.007	0.029	0.001	0.003	0.364	0.002	0.005	0.342
